# Supplementation of tuna hydrolysate and insect larvae improves fishmeal replacement efficacy of poultry by-product in *Lates calcarifer* (Bloch, 1790) juveniles

**DOI:** 10.1038/s41598-021-84660-5

**Published:** 2021-03-02

**Authors:** Md Reaz Chaklader, Janet Howieson, Muhhammad A. B. Siddik, Md Javed Foysal, Ravi Fotedar

**Affiliations:** 1 School of Molecular and Life Sciences, Curtin University, 1 Turner Avenue, Bentley, WA 6102 Australia; 2 Department of Fisheries Biology and Genetics, Faculty of Fisheries, Patuakhali Science and Technology University, Patuakhali, 8602 Bangladesh; 3 Department of Genetic Engineering and Biotechnology, Shahjalal University of Science and Technology, Sylhet, 3114 Bangladesh

**Keywords:** Immunology, Microbiology, Microscopy, Animal physiology

## Abstract

The effects of feeding different levels of poultry by-product meal (PBM) replacing fishmeal (FM) protein, supplemented with tuna hydrolysate (TH) and *Hermetia illucens* (HI) larvae, on the growth, fillet quality, histological traits, immune status, oxidative biomarker levels and gut microbiota of juvenile barramundi, *Lates calcarifer* were investigated for six weeks. Barramundi were fed four isonitrogenous and isolipidic diets in which a FM based diet was used as the Control diet (Diet1) and compared with other non-FM diets containing 80%, 85% and 90% PBM along with the concurrent supplementation of 5% and/or 10% TH and HI larvae meal. These treatment diets were designated as 80PBM_10TH+10HI_ (Diet2), 85PBM_5TH+10HI_ (Diet3) and 90PBM_5TH+5HI_ (Diet4). The growth and condition factor of fish fed 80PBM_10TH+10HI_ and 85PBM_5TH+10HI_ were significantly higher than the Control. Total saturated, monounsaturated and polyunsaturated fatty acid retention in the fish muscle increased in fish fed PBM-based diets, supplemented with TH and HI larvae meal, with no adverse effect on post-harvest characteristics such as texture and colour of fish fillets. Improvement in serum total bilirubin and total protein content was found in all fish fed TH and HI larvae supplemented PBM. Similarly, immune response showed a significant increase in fish fed non-FM test diets than the Control. In the distal intestine, supplementation of any quantities of TH and HI larvae to PBM led to an increase in the microvilli density and neutral mucins while the number of goblet cells in the skin were unchanged. Liver, kidney, and spleen histology demonstrated a normal structure with no obvious changes in response to all test diets. Bacterial diversity increased in fish fed Diets 2 and 3 with a high abundance of *Proteobacteria* in Diets 1 and 4 and *Firmicutes* in Diets 2 and 3. The fish on test diets showed a lower abundance of genus *Vibrio*. Fish fed TH and HI larvae supplemented PBM diets showed lower infection rate to *V. harveyi* than the Control. Collectively, concurrent supplementation of TH and HI larvae could improve the quality of PBM diets with positive effects on growth, fillet quality, intestinal health, immunity, and disease resistance.

## Introduction

Due to a favourable nutritional profile, compatible with the nutritional requirement of most aquaculture species^1^, the aquaculture industry has traditionally relied on fishmeal (FM) as the main protein source^2^. Since this dietary protein source is considered environmentally and ecologically unsustainable, there is societal and economical pressure on the aquaculture industry to search for alternative protein ingredients. Efforts have been dedicated over the past few decades to utilize plant-feedstuffs to replace FM^1^ however the resulting growth performance for carnivorous fish is inferior when compared to FM-based diets, likely due to the lower protein and imbalanced amino acids, anti-nutritional factors, and no taurine and hydroxyproline contents relative to FM in plant ingredients^3–6^.

Rendered animal by-products, particularly poultry by-product (PBM) could be a promising alternative protein source for carnivorous fish due to a higher protein content and lack of anti-nutritional factors^7,8^. The incorporation of 100% PBM replacing FM protein did not affect the welfare of a number of carnivorous fish such as humpback grouper^9^, Nile tilapia, *Oreochromis niloticus*^10,11^ and hybrid striped bass*, Morone chrysops* × *Morone saxatilis*^12^. However, feeding PBM at replacement levels more than 50% impacted the growth performance in barramundi, *L. calcarifer*^13^, Florida pompano, *Trachinotus carolinus* L tench^14^, juvenile tench, *Tinca tinca*^15^*,* black sea turbot*, Psetta maeoticus*^16^ and cobia*, Rachycentron canadum*^17^. In addition, impairment of immunity and serum biochemistry were reported in largemouth bass, *Micropterus salmoides*^18,19^ and barramundi fed PBM^13,20^ and hybrid grouper, *Epinephelus fuscoguttatus*♀ × *Epinephelus lanceolatus*♂ fed an animal protein blend^8^. In our previous studies^13,20,21^, we have also shown that the exclusive inclusion of PBM protein at the expense of FM protein caused histopathological changes in the liver and impaired the integrity of intestinal micromorphology of barramundi. Though a number of studies have been conducted on re-formulating aquadiets supplementing fish protein hydrolysates (FPH)^22–25^ and other supplements^26–28^ that complimented animal proteins and helped to satisfy the needs of barramundi, developing functional sustainable feeds that remain cost-efficient, while promoting fish welfare and maximising growth potential, is a foremost challenge for the aquaculture industry.

FPH due to presence of low molecular weight peptides and free amino acids, have been supplemented in aquafeed as immunostimulants, palatability enhancers and attractants^29–31^. Promising results in terms of growth performance, immune response and disease resistance have been reported for many carnivorous fish species such as barramundi^22,23,32^, Atlantic salmon*, Salmo salar*^33^*,* European sea bass, *Dicentrarchus labrax*^34^ and Persian sturgeon, *Acipenser persicus* L.^35^ when fed FPH at an appropriate level. Moreover, supplementation of FM and PBM with FPH modulated the intestinal microbiome of barramundi by increasing the number of *Bacillus*, *Lactococcus*, and *Cetobacterium* under phylum of *Firmicutes* and *Fusobacteria* and also regulated the inflammatory response^22^.

Recently, insect have received much attention as an aquafeed ingredient due to rapid growth, and ease of reproduction^36^. Insects may be grown on low-value products and the final waste (frass) can be used as organic fertilizer, and insect production also has a lower risk of transmitting zoonotic infections, less greenhouse gas and ammonia emissions and less requirement for land and water than plant-feedstuffs production^6,37–39^. Among all insects investigated so far, the larvae of *Hermetia illucens* (HI), commonly known as black soldier fly has been identified as a potential aquafeed ingredient due to the ability to assimilate nutrients from low-value wastes and by-products and transform such ingredients into high-quality animal biomass, representing a sustainable way to produce edible proteins for livestock production^2,40,41^. It is also noteworthy that the lipid content of HI larvae is highly variable, depending on the substrate used for their culture^42^ and easy to manipulate. Also, HI larvae as an ecological decomposer, dwelling in a harsh environment, infested with a high concentration of harmful microorganisms, can produce novel antimicrobial peptides (AMP) to gain protection from microbial infection^43,44^. Indeed, a new defensin-like peptide4 AMP of 40 amino acids purified from immunized haemolymph of HI larvae has exhibited bactericidal activity to Gram-positive bacterial strains^45^. Hence, the dietary supplementation of HI larvae in the aquadiets could also play an important role in modulating immune response and defensive mechanisms against microbial pathogens.

Among all fish microbiota, bacteria are dominant in the fish intestine^46^. However, the bacterial composition is diverse in different parts of the intestine^47^ and is influenced by diet, life stage, sex, habitat and season^48–52^. Dietary manipulation of protein and lipid also has a selective pressure on the intestinal bacterial diversity in the digestive tract of fish^46,53^. Over the past few decades, many conventional methods have been applied to analyse the gut microbiota of fish^54^ but recently with the development of molecular techniques such as next-generation sequencing (NGS), a rapid and low-cost technique, research on the gut microbiota of fish has evolved rapidly^46^. Like humans and other mammals, there is little doubt concerning the importance of the intestinal microbiota for fish health^22,55,56^, however, dysbiosis of the intestinal microbiota could also be related to growth impairment, dysregulation of immune functions and disease development in fish^57,58^. A recent study^22^, has reported the modulatory effects of alternative protein sources on the intestinal microbiota in barramundi but possible links between diets, growth, and gut functions is fragmentary and incomplete. Hence, the profiling of gut microbiota is expected to be an important endpoint measurement to assess the effects of aquadiet in relation to growth and other health aspects.

As alternative protein ingredients could adversely affect the marketability of fish by altering fillet characteristics, a research evaluating the potential effects of extensive replacement of FM with other animal by-products on the post-harvest quality of fish flesh is highly desirable. Animal by-products particularly meat meal did not alter the organoleptic quality of barramundi fillet^59^, however, excessive inclusion of PBM negatively influenced the fillet quality of female tenches, *Tinca tinca*^60^. Nonetheless, the effects of animal based-diets at the exclusion of FM on the post-harvest quality of barramundi fillet have rarely been investigated with mixed results.

Barramundi is a commercially important euryhaline species, popularly cultured in South East Asian countries and Australia due to economic value and favourable flesh texture. The disease particularly Vibriosis, caused by *V. harveyi* is one of the common problems, causing mortalities in net-cages culture. A number of earlier studies have been conducted so far supplementing plant herbs^27^, FPH^22,24^ and HI larvae^13^ to improve the health status of barramundi. However, concurrent effects of TH and HI larvae meal have not been reported to date. Hence, the present study was designated to investigate the effects of total substitution of dietary FM with three different protein ingredients PBM and, TH and HI larvae meal on gut microbiota, health indices and fillet quality of barramundi. Protein from PBM was the main source of protein whereas TH and HI were supplemented in three different proportions to evaluate the replacement efficiency of PBM protein.

## Materials and methods

### Ethical statement

All experimental protocols involving handling and treatment of animals described in the present study were approved by the Animal Ethics Committee at Curtin University under Permit no. ARE2018-37 in full compliance with relevant guidelines and regulations in Australia for the care and use of animals. To minimize pain and discomfort during handling and culling of fish, anaesthesia (AQUI-S, 8 mg/l) and euthanasia (AQUI-S, 175 mg/l) were applied following standard operating procedure (SOP) of the Curtin Aquatic Research Laboratory (CARL).

### Experimental diets

All the ingredients (Table 1) excluding PBM, TH, and HI larvae were bought from Specialty Feeds, Glen Forrest Stockfeeders, 3150 Great Eastern Highway, Glen Forrest, Western Australia 6071. PBM was provided by Derby Industries Pty Ltd T/A, Talloman Lot Lakes Rd, Hazelmere WA 6055 and liquid TH was provided by SAMPI, Port Lincoln, Australia. Four animal protein based-test diets with similar proximate compositions were formulated (Table 1). FM protein is substituted mainly by PBM followed by supplementing TH and/or HI. The control diet was formulated using FM as a protein source and the other three diets were formulated to replace 80%, 85% and 90% of FM protein with PBM along with the supplementation of 10% TH + 10% HI, 5% TH + 10% HI, and 5% TH + 5% HI, respectively. The four diets were designated as Control (Diet1), 80PBM_10TH+10HI_ (Diet2), 85PBM_5TH+10HI_ (Diet3) and 90PBM_5TH+5HI_ (Diet4). The diets were formulated, packed and stored according to the common standard protocol in our laboratory^13^. Fatty acid and amino acid composition of experimental diets and different ingredients are presented in Tables 2 and 3, respectively.

**Table 1:** Ingredients and nutritional composition of four experimental diets (% dry matter basis).

Ingredients (g/100 g)	Diet1 (Control)	Diet2 (80PBM_10TH+10HI_)	Diet3 (85PBM_5TH+10 HI_)	Diet4 (90PBM_5TH+ 5HI_)
^†^FM^a^	72.6	0.00	0.00	0.00
^††^PBM^b^	0.00	57.00	60.50	63.50
Canola oil^a^	2.20	4.00	4.00	4.00
Cod Liver Oil^a^	1.40	2.50	2.50	3.60
Corn/wheat starch^a^	6.7	8.00	10.00	10.80
wheat (10 CP)^a^	13.5	7.90	5.90	6.00
Lecithin – Soy (70%)^a^	2.00	2.00	2.00	2.00
Vitamin C^a^	0.05	0.05	0.05	0.05
Dicalcium Phosphate^a^	0.05	0.05	0.05	0.05
Vitamin and mineral premix ^a^	0.50	0.50	0.50	0.50
Salt (NaCl)^a^	1.00	1.00	1.00	1.00
^†††^HI full-fat larvae^c^	0.00	10.00	10.00	5.00
^‡^TH^d^	0.00	7.00	3.50	3.50
Total	100	100	100	100
**Nutritional composition (%)**				
Dry matter	92.90	90.35	91.35	92.14
Crude protein	46.20	45.92	46.20	46.72
Crude lipid	12.93	13.01	12.88	13.00
Ash	12.78	12.64	11.89	12.44
Gross energy (MJ kg^-1^)	20.02	19.98	19.88	19.46

^a^Purchased from Specialty Feeds, Glen Forrest Stockfeeders, 3150 Great Eastern Highway, Glen Forrest, Western Australia 6071.

^†^Fishmeal (FM): 64.0% crude protein, 10.76% crude lipid and 19.12% ash.

^††^Poultry by-product meal (PBM): 67.13% crude protein, 13.52% crude lipid and 13.34% ash.

^†††^^†^HI (*Hermetia illucens*) larvae: 40.04% crude protein, 28.30% crude lipid, 7.81% moisture and 8.91% ash.

^‡^Tuna hydrolysate (TH): 37.91% crude protein, 5.50% crude lipid, and 11.05% ash.

**Table 2:** Amino acid composition (g/100 g DM) of PBM-based diets, supplemented with TH and HI larvae meal and different ingredients including PBM, TH and HI larvae meal.

	Diet1 (Control)	Diet2 (80PBM_10TH+10HI_)	Diet3 (85PBM_5TH+10 HI_)	Diet4 (90PBM_5TH+ 5HI_)	PBM	TH	HI
Hydroxyproline	1.79	3.14	3.16	3.37	3.48	1.81	0.24
Histidine	3.01	2.33	2.36	2.27	2.30	2.96	3.27
Taurine	0.48	0.59	0.56	0.53	0.44	2.84	1.08
Serine	4.50	4.22	4.26	4.19	4.34	4.55	4.48
Arginine	6.31	6.89	6.95	6.98	7.32	5.55	5.45
Glycine	7.99	9.62	9.74	9.98	10.13	8.79	5.86
Aspartic acid	9.45	8.84	8.71	8.81	8.50	9.07	10.42
Glutamic acid	13.90	14.55	14.31	14.49	13.83	11.69	13.42
Threonine	4.74	4.13	4.17	4.10	4.19	4.74	4.35
Alanine	6.72	6.65	6.65	6.66	6.61	7.01	6.59
Proline	5.67	6.70	6.70	6.71	6.63	5.64	6.24
Lysine	7.32	6.63	6.56	6.55	6.58	6.98	7.10
Tyrosine	2.87	2.90	2.86	2.79	2.97	2.90	5.97
Methionine	2.92	2.15	2.13	2.15	2.23	2.71	2.02
Valine	5.33	5.02	5.08	4.95	4.92	5.80	6.40
Isoleucine	4.54	4.20	4.24	4.14	4.07	4.61	4.86
Leucine	8.04	7.36	7.45	7.30	7.37	7.88	7.59
Phenylalanine	4.42	4.08	4.12	4.03	4.09	4.49	4.67

**Table 3:** Fatty acids composition (g/100 g DM) of PBM-based diets, combinedly supplemented with TH and HI larvae meal and different ingredients including PBM, TH and HI.

	Diet1 (Control)	Diet2 (80PBM_10TH+10HI_)	Diet3 (85PBM_5TH+10 HI_)	Diet4 (90PBM_5TH+ 5HI_)	PBM	TH	HI
C12:0	0.03	5.02	5.23	2.44	0.09	0.04	53.81
C14:0	1.32	2.10	1.96	1.66	0.74	4.75	8.87
C16:0	11.61	18.13	17.73	17.01	23.36	28.56	20.47
C18:0	4.49	5.73	5.55	5.34	7.62	10.92	4.65
**∑SFA**	19.81	33.03	32.41	28.13	33.45	48.41	93.05
C14:1n5	0.02	0.10	0.10	0.09	0.16	0.04	0.15
C16:1n7	1.65	3.93	3.90	3.71	5.40	5.70	6.21
C18:1cis + trans	11.59	43.54	43.93	42.49	44.11	33.34	23.37
C20:1	0.80	2.03	1.96	2.34	0.61	2.56	0.29
C22:1n9	0.09	0.20	0.19	0.25	0.06	0.31	0.00
**∑MUFA**	14.82	50.41	50.64	49.47	50.42	43.01	30.67
C18:3n3	1.20	4.28	4.37	3.99	2.60	1.53	5.17
C18:4n3#	0.30	0.55	0.47	0.42	0.13	2.25	1.84
C20:3n3	0.08	0.08	0.07	0.07	0.04	0.25	0.11
C20:5n3	1.78	2.15	1.68	1.77	0.17	12.12	3.67
C22:5n3#	0.63	0.66	0.53	0.60	0.37	3.41	0.28
C22:6n3	9.09	4.25	2.95	3.49	0.27	32.32	0.78
**∑n-3 PUFA**	13.09	11.97	10.06	10.34	3.59	51.88	11.85
**∑n-3 LC PUFA**	11.59	7.14	5.22	5.93	0.01	48.10	4.83
C20:2	0.15	0.18	0.17	0.17	0.20	0.35	0.12
C18:3n6	0.09	0.09	0.09	0.13	0.23	0.21	0.15
C20:3n6	0.15	0.30	0.30	0.30	0.57	0.47	0.23
C20:4n6	1.13	1.01	0.98	0.93	1.80	1.99	1.31
C22:4n6#	0.91	0.17	0.12	0.13	0.05	1.18	0.20
**∑n-6 PUFA**	2.28	1.58	1.50	1.49	2.65	3.86	1.88
**∑PUFA**	21.84	31.24	29.86	29.00	23.87	58.32	27.38

### Experimental fish and feeding

The feeding trial was conducted in a recirculating aquaculture system at CARL, Curtin University, Australia. A total of 450 juvenile barramundi (mean weight, 3.33 g) were provided by the Australian Centre for Applied Aquaculture Research, Fremantle, Australia and shipped to CARL in double-layered plastic bags filled with oxygenated 30 ppt saline water. Fish were housed in three 300L fiberglass tanks containing seawater for two weeks to adapt to the CARL conditions. Throughout the acclimation period, one-third of the water was changed daily and fish were hand-fed with a commercial diet. Fish were fasted for 24 h before commencing the trial and then 300 fish with almost similar weights were distributed randomly into 12 tanks (4 dietary treatments × 3 replicated tanks), with 25 individuals in each tank. Water quality parameters including temperature, dissolved oxygen, nitrite, nitrate, and temperature controlled by an electric heater, aerator and external bio-filter (ASTRO 2212, China), were maintained as described in our earlier study^13^. 14:10 h light: dark photoperiod was maintained throughout the study period using an automatic indoor timer (Clipsal, Australia). Fish were hand-fed the experimental diets until apparent satiation, twice daily at 8.00 am and 6.00 pm for 6 weeks. After each feeding session, provided feed and uneaten feed weights were recorded to calculate FI on a daily basis. Fish health and mortality, if any, were monitored regularly to calculate the survival rate.

### Sampling procedure

After the six weeks of feeding trial, fish were starved for 24 h and anesthetized with 8 mg/l AQUI-S prior to taking total biomass, and fish were also counted individually to estimate survival rate. Viscera and liver weight were recorded from randomly chosen ten fish from each treatment to determine viscerosomatic (VSI) and hepatosomatic (HSI) indices. For serum collection, four fish from each tank (n = 12) were anesthetized with 8 mg/l AQUI-S and blood was collected from the caudal vein using 1 mL syringes fitted with 22G needles. Serum was collected and preserved according to the protocols of our earlier study^13^. Fish were then culled with a sharp blow to the head and dissected on trays to collect muscle, liver, and intestine samples. Pooled “fillet” muscle was collected and dried in a freeze dryer and stored at -80^◦^ C for further fatty acid analysis. Also, four fish/replicate were filleted to analyse texture and colour. The liver and intestine were also preserved immediately in -80^◦^ C until later analysis of antioxidant activity and gut microbiota. Four fish per tank were also euthanized to collect intestine, muscle with skin, liver, kidney, and spleen and immediately preserved in 10% buffered formalin for fixation for later histological examination.

### Fillet fatty acids compositions and quality index

Fatty acids profile of four pooled dried muscle/replicate and experimental diets were performed according to the procedures of O'Fallon, et al.^61^ and Siddik, et al.^21^.

Texture profile in terms of hardness, cohesiveness, adhesiveness, springiness, gumminess, chewiness of fish fillet (three fish/treatment) were analysed by texture analyser TVT 6700 (PerkinElmer, Inc., Waltham, Middx, USA) and TexCal texture analyser software (version 5.0)^62^. Each sample was run in triplicate and expressed as force per g. Fillet colour coordinates (L*, a*, b*) were determined by calibrated BYK spectro-guide sphere gloss (BYK-Gardener USA, Columbia, MD, USA)^63^. 4 g of fillet were homogenized with 40 mL of distilled water and pH was determined in triplicate by Aqua-pH meter (TPS Pty Ltd, Brendale, QLD, AU) after calibrating at three-scale pH (pH 4, 7, 10)^64^.

### Histological and scanning electron microscopy analysis

After fixation with 10% buffered formalin, fragments of intestine, liver, kidney, spleen, and skin tissue samples were dehydrated with a series of ethanol concentrations. Dehydrated samples were subsequently cleared in xylene, embedded in paraffin blocks, cut into around 5 µm slices, and stained with haematoxylin and eosin (H&E) for histopathological assessment. Intestine and liver samples were stained with Periodic Acid-Schiff (PAS) to visualize neutral mucins and glycogen, respectively. Skin tissue was stained with AB-PAS stain to visualize goblet cells. Microphotographs of all histological slides were taken with a light imaging microscope (BX40F4, Olympus, Tokyo, Japan). The number of neutral mucins in intestine and skin was counted as described in our earlier study^13^.

Scanning electron microscopy of distal intestine from four biological replicates were analysed according to the earlier study in our laboratory^24^. Intestinal samples (5 mm) were washed for 30 s with 1% S-carboxymethyl-L-cysteine to remove mucus and then fixed in 2.5% glutaraldehyde in sodium cacodylate buffer (0.1 M pH 7.2). Samples were processed as described elsewhere, screened with JSM 6610 LV (Jeol, Tokyo, Japan) SEM and analysed with Image J 1.46r (National Institute of Health, USA).

### Serum immunity and biochemical assays

Serum immunity including lysozyme and bactericidal activity were analysed as described in our earlier study^13^.

The plasma clinical chemistry panel was processed on a AU480 Clinical Chemistry Analyser (Beckman Coulter Australia Pty Ltd, Lane Cove West, NSW). Beckman Coulter clinical chemistry kits were used for the following panel components: alanine transaminase (ALT OSR6107) gamma-glutamyl transferase (GGT OSR6219) total bilirubin (TB OSR6112), urea (OSR6134), creatinine (OSR6178), cholesterol (OSR6116), total protein (TP OSR6132) while Randox kits (Randox Australia Pty Ltd, Parramatta, NSW) were used for glutamate dehydrogenase (GLDH GL441).

### Serum and liver antioxidant activity

For each replicate/treatment, approximately 0.20 mg of liver tissues was weighed and homogenized with 2 mL of chilled PBS. Immediately homogenized tissue was centrifuged at 10,000 × g for 15 min at 4^◦^ C and supernatant was collected and stored at 80^◦^ C till analysis.

Catalase activity (CAT) in serum and liver homogenate were performed according to the manufacturer company instruction (Bockit, BIOQUOCHEM SL, 33,428 Llanera-Asturias, Spain).

## Microbiome analysis

### Amplicon sequencing

After the trial, two randomly selected fish per tank (n = 6) were used for gut microbiota analysis. Processing of samples comprised of a collection of the whole gut, separation of the hindgut, homogenization of gut contents in tissue lyserII (Qiagen, Hilden, Germany), and pooling of homogenized samples from the respective tanks (n = 3). Bacterial DNA from pooled the hindgut samples was extracted using Blood and Tissue Kit (Qiagen, Hilden, Germany). DNA concentration was measured in NanoDrop (Thermo Fisher Scientific, USA). PCR master mixture was prepared as 50 µl final concentration containing 25 µl Hot Start 2X Master mix (New England BioLabs Inc., USA), 2 µl of sample DNA, 1 µl of each V3V4 primers^65^ and 21 µl of nuclease-free water. A total of 40 cycles of amplification reactions was performed in a thermal cycler (Bio-Rad Laboratories, Inc., USA). The library for 16S rRNA amplicons was prepared according to Illumina standard protocol (Part # 15,044,223 Rev. B). Samples were then sequenced on an Illumina MiSeq platforms (Illumina Inc., San Diego, California, USA using a v3 kit (600 cycles).

### Sequence data processing and analysis

Low quality reads (phred score ≤ 20, > 6 homopolymers), adaptors, short bases (< 200 bp) were trimmed and cleaned using TrimGalore (http://www.bioinformatics.babraham.ac.uk/projects/trim_galore/). The quality of sequences was checked before and after trimming in FastQC and MultiQC pipelines^66,67^. MeFiT program was used for the merging of overlapping pair-end reads^68^. De novo assembly, picking of OTUs at 97% similarity threshold level and removing of singleton OTUs were performed in micca (v1.6.1)-qiime (v2.0) pipelines^69,70^. Phylogenetic assignment of OTUs at different taxa levels was performed against SILVA 1.32 release^71^. PASTA-aligned sequences were used for phylogenetic tree constructions under FastTree (version 2.1.8) GTR + CAT^72,73^. The rarefaction depth was set to 38,296 bp and subsequent measurements of alpha–beta diversity were performed using qiime (v1.9.1) and R packages. Alpha diversity was calculated in terms of observed OTUs and Shannon indices. Beta diversity was measured based on Bray–Curtis dissimilarity of Weighted UniFrac matric. Relative abundance (≥ 1% of read abundance) of phyla and genera was calculated phyloseq R package^74^. Differential abundance was calculated using the linear discriminant analysis effect size (LEfSe) at LDA ≥ 2.0 and 0.05 level of significance^75^.

### Challenge test

After the feeding trial, 10 fish/tank (30 fish/treatment) with the same rearing condition were challenged with *V. harveyi*, provided by Diagnostic and Laboratory Services, Department of Primary Industries and Regional Development (DPIRD), 3 Baron-Hay Court, South Perth WA 6151. Fish were intraperitoneally injected with 0.1 mL of phosphate-buffered saline (PBS) containing 1.1 × 10^8 cfu/ml of a pathogenic strain of *V. harveyi* as elucidated in our earlier study^25^. Symptoms of vibriosis such as thick layer of mucous on the body surface, congestion of the fins, and haemorrhages and ulceration of the skin and muscle tissue were monitored every 8 h and lasted for 96 h. Fish with vibriosis were culled according to the protocol of the CARL SOP for fish euthanization.

### Calculation and statistical analysis

Weight gain (WG), specific growth rate (SGR), feed intake (FI), feed conversion ratio (FCR), condition factor (CF), viscerosomatic index (VSI) and hepatosomatic index (HSI) were estimated using the following formulae:

$${\text{Weight gain }}\left( {{\text{WG}},{\text{ g}}} \right) = \left[ {\left( {{\text{Mean final weight}} - {\text{Mean initial weight}}} \right)/\left( {\text{Mean initial weight}} \right)} \right]$$

$${\text{Specific growth rate }}\left( {{\text{SGR}},{{ \% }} / {\text{d}}} \right) = \left[ {\left( {{\text{ln }}\left( {\text{ final body weight}} \right) - {\text{ln }}\left( {\text{pooled initial weight}} \right)} \right)/{\text{Days}}} \right] \times 100$$

$${\text{Feed intake }}\left( {{\text{FI}},{\text{g}}/{\text{fish d}}^{ - 1} } \right) = \left[ {\left( {{\text{Dry diet given}} - {\text{Dry remaining diet recovered}}} \right)/{\text{days of experiment}}} \right)/{\text{ no}}.{\text{ of fish}}]$$

$${\text{Feed conversion ratio }}\left( {{\text{FCR}}} \right) = { }\left[ {\left( {\text{Dry feed fed}} \right)/\left( {\text{Wet weight gain}} \right)} \right]$$

$${\text{Condition factor }}\left( {{\text{CF}},{{ \% }}} \right) = { }\left[ {{\text{Final body weight }}\left( {\text{g}} \right)/{\text{Body length cm}}^{3} { }} \right)] \times 100$$

$${\text{Hepatosomatic index }}\left( {{\text{HSI}},{{ \% }}} \right) = { }[({\text{Liver weight }}\left( {\text{g}} \right)/\left( {{\text{Whole body weight }}\left( {\text{g}} \right)} \right] \times 100$$

$${\text{Viscerosomatic index }}\left( {{\text{VSI}},{{ \% }}} \right) = { }\left[ {{\text{Viscera weight }}\left( {\text{g}} \right)/{\text{Whole body weight }}\left( {\text{g}} \right)} \right] \times 100$$

All experimental results are presented as mean ± standard error (SE). Groups of fish per tank were used as experimental units for growth data. Individual fish were used as experimental units for organo-somatic assessment, biochemical assays, immune response, fatty acids, antioxidant activity, and histological analysis. One-way analysis of variance (ANOVA) with Dunnett’s multiple comparisons test was performed to the significant differences between treatments when data met the normality, checked by Shapiro–Wilk's and Levene's tests. Infection data from the challenge trial were analysed by the Kaplan–Meier method based on the pairwise multiple comparison Log-Rank (Mantel-Cox) test.

## Results

### Growth performance and organo-somatic indices

The effects of different levels of PBM concurrently supplemented with TH and HI on the growth performance and organo-somatic indices are shown in Table 4. Fish fed Diet2 (80PBM_10TH+10HI_) and Diet3 (85PBM_5TH+10HI_) recorded significantly higher final body weight (FBW), weight gain (WG) and specific growth rate (SGR) after six weeks of feeding whereas fish fed Diet 4 (80PBM_5TH+5HI_) was similar to Control. No significant variation was observed in feed intake (FI) and feed conversion ratio (FCR) between Control and test diets. The survival rate, ranging from 89.33 to 93.33%, was not influenced by the test diets. Condition factor (CF) increased significantly in fish fed supplemented TH and HI larvae diets, although hepatosomatic (HSI) and viscerosomatic (VSI) indices were not influenced by any test diets.

**Table 4:** Growth performance, feed utilization, survival and organo-somatic indices of barramundi fed Control (Diet1), 80PBM_10TH+10HI_ (Diet2), 85PBM_5TH+10HI_ (Diet3), and 90PBM_5TH+5HI_ (Diet4) for six weeks.

	Diet1 (Control)	Diet2 (80PBM_10TH+10HI_)	Diet3 (85PBM_5TH+10 HI_)	Diet4 (90PBM_5TH+ 5HI_)
FBW (g)	50.05 ± 2.05^c^	67.60 ± 1.93^a^	60.49 ± 2.37^ab^	54.24 ± 2.57^bc^
WG (g)	42.30 ± 2.05^c^	59.84 ± 1.93^a^	52.73 ± 2.37^ab^	46.48 ± 2.57^bc^
SGR (%/d)	4.28 ± 0.11^c^	5.07 ± 0.08^a^	4.73 ± 0.11^ab^	4.42 ± 0.13^bc^
FI (g/fish/d)	1.48 ± 0.10	1.60 ± 0.11	1.40 ± 0.19	1.40 ± 0.17
FCR	1.49 ± 0.16	1.14 ± 0.07	1.11 ± 0.05	1.28 ± 0.08
SR (%)	90.67 ± 3.53	89.33 ± 1.33	93.33 ± 1.33	92.00 ± 2.31
CF (%)	1.27 ± 0.01^b^	1.38 ± 0.01^a^	1.35 ± 0.01^a^	1.37 ± 0.01^a^
VSI (%)	9.86 ± 0.46	10.69 ± 0.40	11.06 ± 0.30	11.08 ± 0.30
HSI (%)	1.70 ± 0.07	1.75 ± 0.08	1.67 ± 0.07	1.77 ± 0.08

Multiple comparisons were performed by one-way ANOVA analysis followed by Dunnett’s multiple comparisons test.

Results are expressed as mean ± SE (standard error) (n = 3). Means with different superscripts letters in the same row indicates significant difference at *P* < 0.05.

### Muscle chemical and fatty acid composition

Moisture and ash were unchanged among the test diets at the end of the trial. Lipid in muscle of fish fed PBM based-diets supplemented with TH and HI larvae increased significantly while protein levels decreased significantly in PBM based diets (Table 5). The concentration of C12:0, C14:0, C16:0, and C18:0 led to an increase in the total SFA in the muscle of fish fed PBM-based diets, concurrently supplemented with TH and HI larvae. Total MUFA content increased in the same diets and it was mainly due to the higher concentration of C14:1n5, C16:1n7, C20:1, C18:1cis + trans, and C22:1n9. TH and HI larvae supplemented PBM substantially improved the total PUFA, manifested by an elevated level of C18:3n3, C18:4n3, C20:3n3, C22:5n3, C18:3n6, C20:3n6 and C20:4n6. However, C22:6n3 and C22:4n6 decreased significantly in TH and HI larvae supplemented PBM fed fish than the Control diet.

**Table 5:** Chemical composition and fatty acids composition (mg g^-1^ DM) of barramundi muscle (skin less) after six weeks feeding with a gradient PBM diets, concurrently supplemented with TH and HI larvae meal.

	Diet1 (Control)	Diet2 (80PBM_10TH+10HI_)	Diet3 (85PBM_5TH+10 HI_)	Diet4 (90PBM_5TH+ 5HI_)
**Chemical composition (%)**				
Moisture (WW)	75.22 ± 0.37	74.88 ± 0.44	75.67 ± 0.15	75.12 ± 0.33
CP (DM)	75.27 ± 0.18^a^	70.07 ± 0.62^b^	70.72 ± 0.60^b^	71.16 ± 0.42^b^
CL (DM)	5.70 ± 0.13^b^	10.70 ± 0.38^a^	10.58 ± 0.53^a^	10.74 ± 0.25^a^
Ash (DM)	5.69 ± 0.02	5.61 ± 0.13	5.67 ± 0.08	5.65 ± 0.10
**Fatty acid composition, mg g**^**−1**^** DM**				
C12:0	0.03 ± 0.01^c^	1.55 ± 0.09^a^	1.63 ± 0.10^a^	0.74 ± 0.04^b^
C14:0	0.52 ± 0.06^b^	1.18 ± 0.06^a^	1.17 ± 0.06^a^	0.96 ± 0.06^a^
C16:0	5.81 ± 0.31^b^	10.99 ± 0.53^a^	11.13 ± 0.41^a^	10.92 ± 0.40^a^
C18:0	2.00 ± 0.11^b^	3.70 ± 0.15^a^	3.69 ± 0.09^a^	3.63 ± 0.13^a^
**∑SFA**	9.21 ± 0.51^b^	18.46 ± 0.88^a^	18.60 ± 0.68^a^	17.21 ± 0.66^a^
C14:1n5	0.01 ± 0.00^b^	0.04 ± 0.00^a^	0.05 ± 0.00^a^	0.04 ± 0.00^a^
C16:1n7	0.86 ± 0.05^b^	2.27 ± 0.14^a^	2.32 ± 0.10^a^	2.20 ± 0.11^a^
C20:1	0.48 ± 0.04^b^	1.06 ± 0.07^a^	1.06 ± 0.05^a^	1.25 ± 0.05^a^
C18:1cis + trans	7.43 ± 0.45^b^	22.85 ± 1.35^a^	23.61 ± 1.09^a^	23.59 ± 0.85^a^
C22:1n9	0.05 ± 0.01^a^	0.10 ± 0.01^b^	0.10 ± 0.00^b^	0.12 ± 0.01^a^
**∑MUFA**	9.11 ± 0.56^b^	26.65 ± 1.58^a^	27.44 ± 1.26^a^	27.53 ± 1.00^a^
C18:3n3	0.72 ± 0.05^b^	2.03 ± 0.12^a^	2.11 ± 0.10^a^	1.91 ± 0.08^a^
C18:4n3#	0.11 ± 0.01^b^	0.31 ± 0.02^a^	0.28 ± 0.02^a^	0.26 ± 0.02^a^
C20:3n3	0.03 ± 0.00^b^	0.06 ± 0.00^a^	0.07 ± 0.00^a^	0.06 ± 0.00^a^
C20:5n3	0.80 ± 0.03^b^	1.24 ± 0.02^a^	1.06 ± 0.04^a^	1.10 ± 0.07^a^
C22:5n3#	0.47 ± 0.04^b^	0.80 ± 0.04^a^	0.74 ± 0.02^a^	0.69 ± 0.03^a^
C22:6n3	6.26 ± 0.34^a^	4.36 ± 0.09^b^	3.32 ± 0.06^c^	3.73 ± 0.12^bc^
**∑n-3 PUFA**	8.39 ± 0.48	8.78 ± 0.29	7.57 ± 0.23	7.74 ± 0.32
**∑n-3 LC PUFA**	7.56 ± 0.42^a^	6.45 ± 0.16^ab^	5.18 ± 0.12^c^	5.58 ± 0.23^bc^
C18:3n6	0.08 ± 0.01^b^	0.21 ± 0.02^a^	0.26 ± 0.01^a^	0.21 ± 0.01^a^
C20:3n6	0.13 ± 0.01^b^	0.28 ± 0.02^a^	0.33 ± 0.01^a^	0.28 ± 0.01^a^
C20:4n6	0.64 ± 0.03^b^	0.94 ± 0.01^a^	0.93 ± 0.01^a^	0.91 ± 0.02^a^
C22:4n6#	0.52 ± 0.03^a^	0.17 ± 0.00^b^	0.13 ± 0.00^b^	0.13 ± 0.00^b^
**∑n-6 PUFA**	1.37 ± 0.07^b^	1.60 ± 0.04^a^	1.65 ± 0.04^a^	1.53 ± 0.04^ab^
**∑PUFA**	6.73 ± 0.41^b^	15.19 ± 0.65^a^	15.61 ± 0.63^a^	14.68 ± 0.68^a^

Results are expressed as mean ± SE (standard error) (n = 12). Means with different superscripts letters in the same row indicates significant difference at P < 0.05.

### pH, Texture and colour of fish fillet

At the end of the trial, pH, texture profile (springiness, cohesiveness, gumminess, chewiness, adhesiveness and hardness) and colour (L*, a*, b* and chroma) in skin and fillet flesh were similar in fish fed any diet (Table 6).

**Table 6:** Texture profile and colour of barramundi fillet after six weeks feeding with a gradient PBM diets, concurrently supplemented with TH and HI larvae meal.

	Diet1 (Control)	Diet2 (80PBM_10TH+10HI_)	Diet3 (85PBM_5TH+10 HI_)	Diet4 (90PBM_5TH+ 5HI_)
P^H^	6.90 ± 0.05	6.91 ± 0.04	6.90 ± 0.02	6.91 ± 0.02
**Texture parameter**				
Springiness	0.99 ± 0.01	1.00 ± 0.00	0.98 ± 0.01	0.98 ± 0.01
Cohesiveness	0.36 ± 0.03	0.33 ± 0.00	0.35 ± 0.03	0.32 ± 0.01
Gumminess (g)	957.76 ± 82.30	1433.56 ± 165.93	1147.32 ± 149.53	1431.16 ± 115.31
Chewiness (g)	957.53 ±	1430.60 ±	1144.52 ±	1426.03 ±
Adhesiveness (g.mm)	− 13.51 ± 2.17	− 15.82 ± 4.24	− 16.07 ± 2.42	− 21.30 ± 6.93
Hardness	2780.83 ± 462.31	4427.33 ± 477.80	3369.83 ± 525.92	4453.17 ± 494.24
**Skin colour**				
L*	60.93 ± 1.81	59.30 ± 3.58	59.99 ± 1.75	59.99 ± 1.57
a*	− 1.37 ± 0.10	− 1.30 ± 0.24	− 1.20 ± 0.12	− 1.45 ± 0.07
b*	− 4.06 ± 0.26	− 4.03 ± 0.43	− 4.76 ± 0.39	− 3.60 ± 0.25
Chroma	4.30 ± 0.23	4.26 ± 0.44	4.93 ± 0.36	3.90 ± 0.22
**Fillet muscle colour**				
L*	56.55 ± 1.12	57.11 ± 1.03	60.25 ± 1.03	58.61 ± 1.52
a*	0.37 ± 1.07	− 1.04 ± 0.32	− 1.36 ± 0.29	− 1.21 ± 0.56
b*	3.90 ± 1.13	3.72 ± 0.41	4.80 ± 0.59	4.04 ± 0.32
Chroma	4.36 ± 1.32	4.00 ± 0.28	5.08 ± 0.52	4.40 ± 0.34

L*, light/dark; a*, red/green and b*, yellow/blue.

### Histological analysis of liver, kidney, and spleen

**Figure 1:**
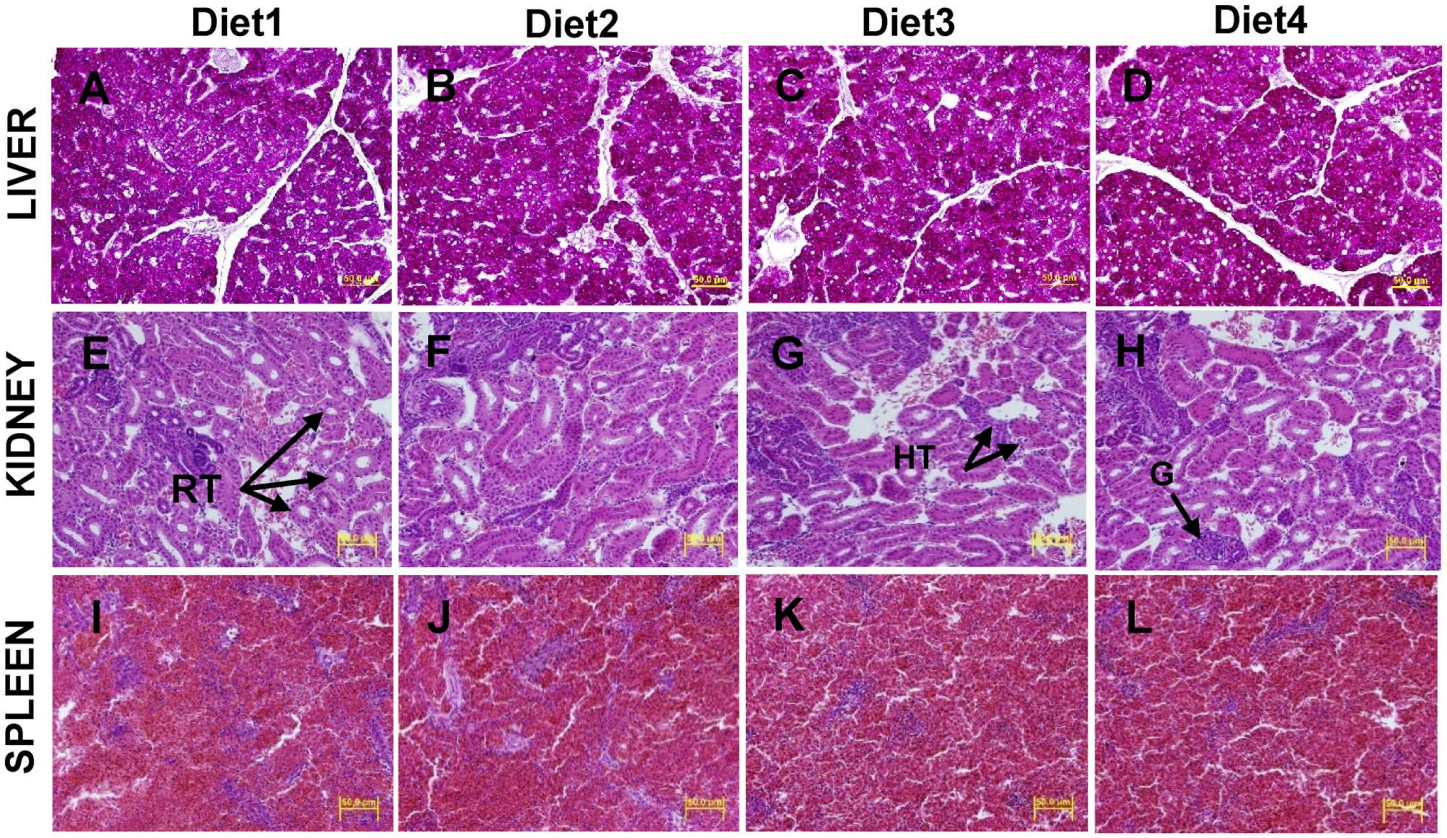
Representative micrographs of staining liver (**A**–**D**), kidney (**E**–**H**) and spleen (**I**–**L**) sections of barramundi fed control (Diet1), 80PBM_10TH+10HI_ (Diet2), 85PBM_5TH+10HI_ (Diet3), and 90PBM_5TH+5HI_ (Diet4) for six weeks using PAS and H&E (40 × magnification). RT, renal tubule; HT, hematopoietic tissue and G, glomerulus

Hitopathological analysis did not demonstrate any substantial changes in the liver, kidney and spleen between diets at the end of the trial (Fig. 1A–L). Higher pigmented hepatic cytoplasm indicating higher amount of glycogen was examined in the liver of fish fed all test diets (Fig. 1A–D). In all dietary group, normal renal glomeruli and renal tubules in the kidney (Fig. 1E–H) and normal white and red pulp in the spleen (Fig. 1I–L) were observed.

### Intestinal mucosal morphology and histochemistry

**Figure 2:**
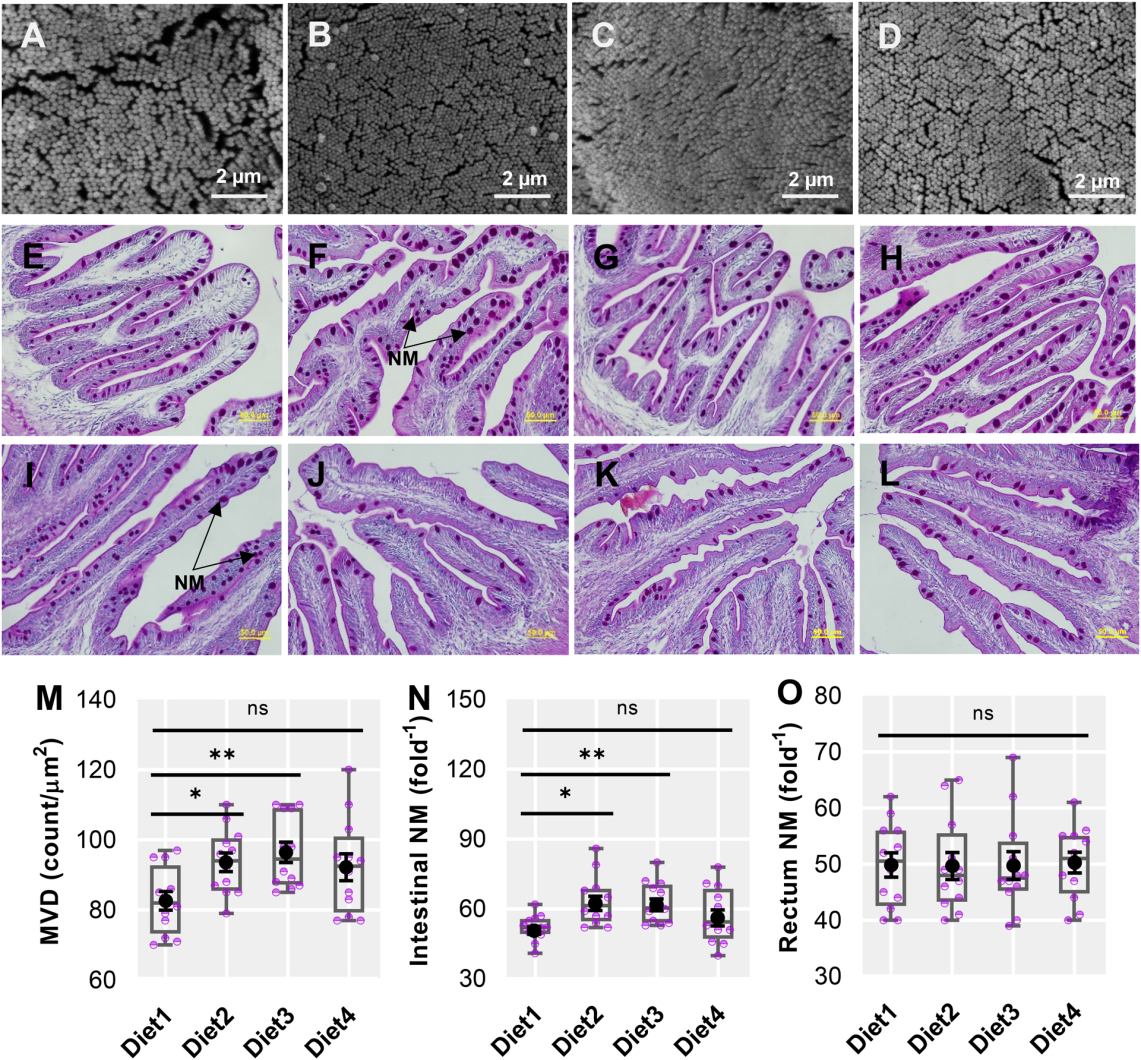
Scanning electron micrographs (**A**–**D**) to visualize the microvilli density and goblet cells containing neutral mucins (**E**–**H**, PAS stain, 40 × magnification) in the distal intestine (**E**–**H**) and rectum (**I**–**L**) of barramundi fed Control (Diet1), 80PBM_10TH+10HI_ (Diet2), 85PBM_5TH+10HI_ (Diet3), and 90PBM_5TH+5HI_ (Diet4) for six weeks. Variation in the microvilli density (M) and neutral mucins in the distal intestine (**N**) and rectum (**O**) of barramundi fed test diets. Multiple comparisons were performed by one-way ANOVA analysis followed by Dunnett’s multiple comparisons test. NM, neutral mucins and ns, not significant.

The intestinal mucosal micromorphology of barramundi fed Control and test diets were examined by SEM (Fig. 2A–D) and light microscopy (Fig. 2E–L). Microvilli count (density) (Fig. 2M) and neutral mucins (Fig. 2N) were significantly higher in barramundi fed TH and HI larvae supplemented at different levels of PBM when compared with those fed the Control diet. However, neutral mucins producing goblet cells in rectum were unchanged (Fig. 2O).

### Skin histochemistry

**Figure 3:**
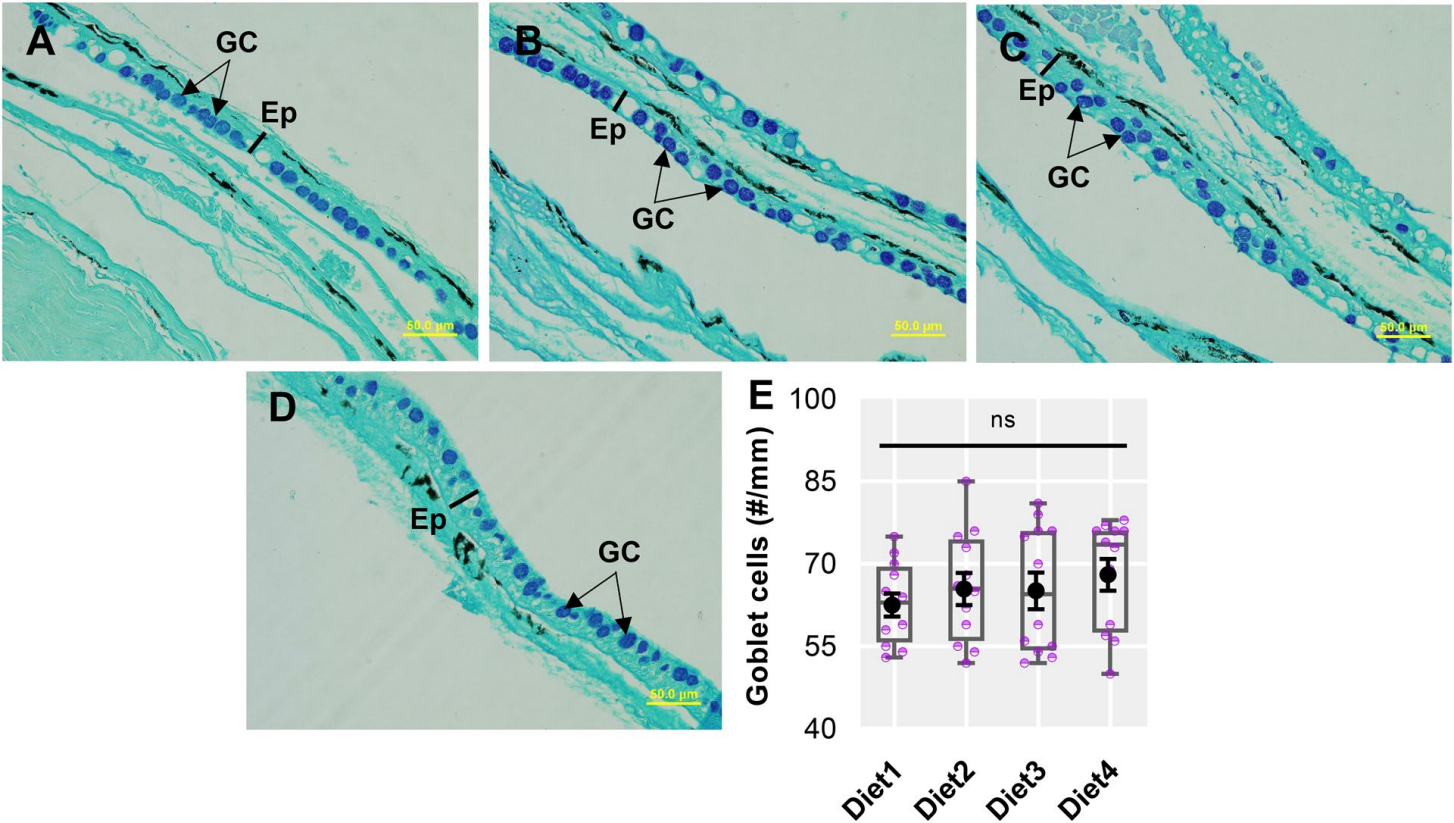
Representative skin micrographs (**A**–**D**, AB-PAS stain, 40 × magnification) and number of mixed goblet cells of barramundi fed Control (Diet1), 80PBM_10TH+10HI_ (Diet2), 85PBM_5TH+10HI_ (Diet3), and 90PBM_5TH+5HI_ (Diet4) for six weeks. Variation in the mixed types of goblet cells in the skin (**E**) of barramundi fed test diets. Multiple comparisons were performed by one-way ANOVA analysis followed by Dunnett’s multiple comparisons test. ns denotes not significant. GC, goblet cell and Ep, epidermis.

The results of skin morphology in response to different levels of PBM, supplemented with TH and HI larvae are shown in Fig. 3A–E. Mixed types of goblet cells were unchanged in fish fed Control and test diets (Fig. 3E).

### Biochemical assays, immune response and antioxidant activity

A significant decrease in the total bilirubin activity of fish fed PBM diets supplemented with TH and HI larvae meal was observed, whilst total protein content in fish fed 85PBM_5TH+10 HI_ increased significantly when compared to the Control (Table 7). The remaining biochemical assays including AST, GGT, GLDH, urea, creatinine, and cholesterol were not affected by any of the test diets. Concurrent supplementation of TH and HI larvae meal to PBM diets also modulated the serum lysozyme and bactericidal activity than Control. CAT activity both in serum and liver was not affected by different levels of PBM supplemented with TH and HI larvae meal with respect to the Control. Liver protein content did not differ significantly among the test diets.

**Table 7:** Biochemical assays, immune response and antioxidant activity of barramundi fed with a gradient PBM diets, concurrently supplemented with TH and HI larvae meal for six weeks.

	Diet1 (Control)	Diet2 (80PBM_10TH+10HI_)	Diet3 (85PBM_5TH+10 HI_)	Diet4 (90PBM_5TH+ 5HI_)
**Serum biochemical assays**				
ALT (U/L)	2.00 ± 1.00	3.67 ± 0.88	3.33 ± 1.33	8.33 ± 1.67
GGT (U/L)	0.00 ± 0.00	0.67 ± 0.33	0.67 ± 0.33	0.67 ± 0.33
GLDH (U/L)	8.00 ± 0.57	6.00 ± 0.00	6.67 ± 0.33	6.33 ± 0.88
Total Bilirubin (umol/L)	3.33 ± 0.33^a^	2.00 ± 0.00^b^	2.00 ± 0.00^b^	2.00 ± 0.00^b^
Urea (mmol/L)	4.07 ± 0.47	5.47 ± 0.59	5.50 ± 0.12	5.90 ± 0.28
Creatinine (umol/L)	17.33 ± 0.88	21.00 ± 1.00	19.00 ± 0.00	18.67 ± 0.33
Cholesterol (mmol/L)	8.08 ± 0.16	13.08 ± 1.24	10.77 ± 0.37	11.38 ± 1.43
Total Protein (g/L)	43.47 ± 0.52^b^	49.07 ± 1.47^ab^	47.60 ± 0.90^a^	45.87 ± 0.22^b^
**Serum immune response and antioxidant activity**				
Lysozyme (U/mL)	765.56 ± 85.69^bc^	1025.56 ± 76.60^a^	940.22 ± 20.76^ab^	668.89 ± 66.45^c^
BA (log_10_)	4.60 ± 0.26^a^	2.50 ± 0.30^b^	2.45 ± 0.64^b^	3.76 ± 0.68^ab^
CAT (U mg.prot^-1^)	0.82 ± 0.32	0.74 ± 0.12	0.72 ± 0.22	0.56 ± 0.06
**Liver protein and antioxidant activity**				
Total protein (mg/mL)	3.87 ± 0.59	2.69 ± 0.07	2.34 ± 0.32	3.04 ± 0.17
CAT (U mg.prot^-1^)	786.33 ± 72.76	692.67 ± 146.68	917.00 ± 178.18	757.33 ± 196.17

Results are expressed as mean ± SE (standard error) (n = 3). Means with different superscripts letters in the same row indicates significant difference at P < 0.05.

Alanine transaminase, ALT; gamma-glutamyl transferase, GGT; glutamate dehydrogenase, GLDH, bactericidal activity, BA and catalase activity, CAT.

### Sequence stats and alpha–beta diversity

**Figure 4:**
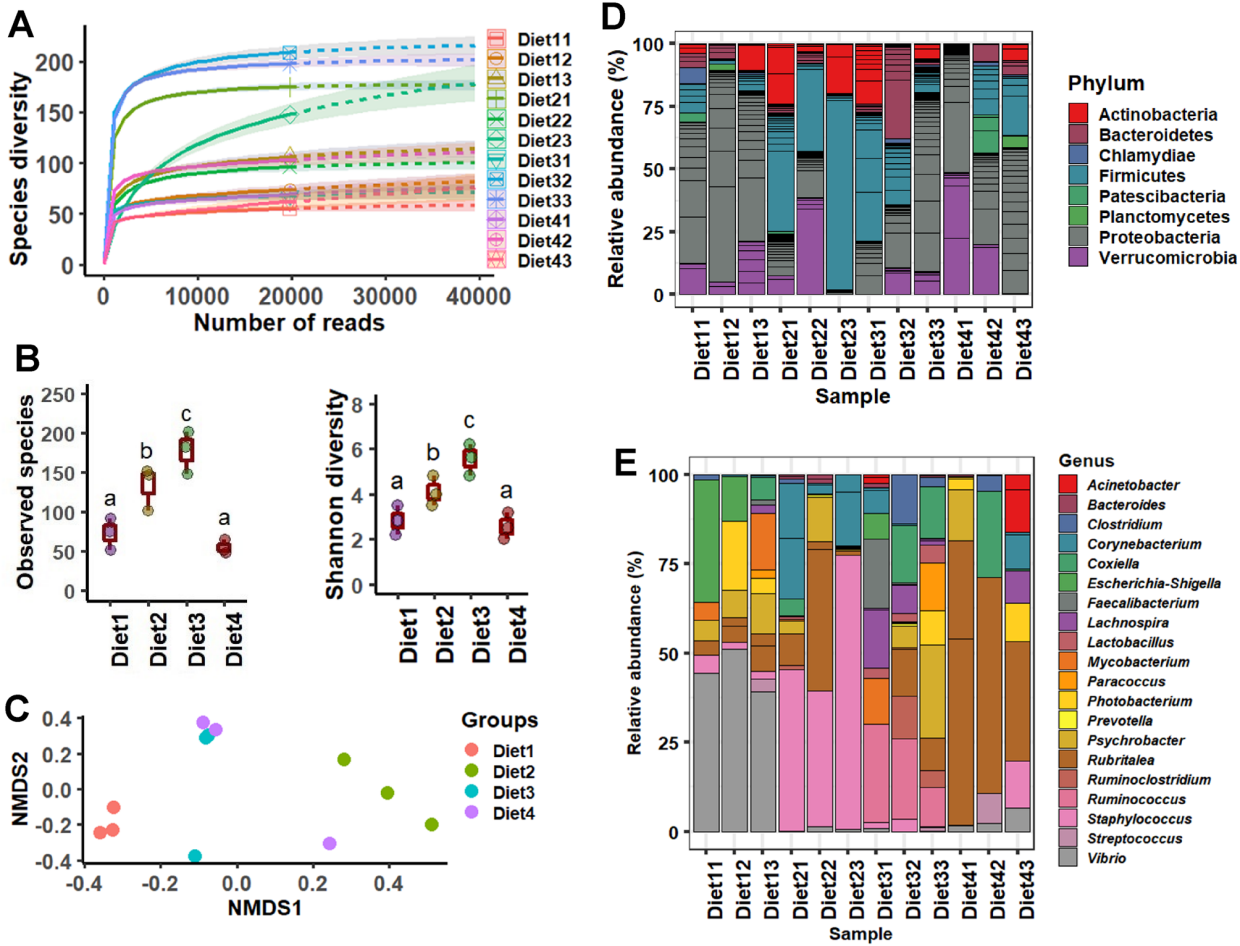
Rarefaction curve (**A**) showing the depth of sequencing in terms of species diversity, observed species and Shannon diversity (**B**), Non-metric multidimensional scaling (nMDS) plot demonstrating clustering (**C**) and relative abundance of bacteria at phylum (**D**) and genus (**E**) level of different test diets fed to barramundi for 6 weeks.

Quality trimming yielded total of 537,070 reads, ranging from 38,180 to 57,975 (44,755.8 ± 28,444.2), extracted from amplicon sequence data that assigned into 415 OTUs (286.8 ± 48.2), 19 phyla and 176 genera. The highest average OTUs (328.6 ± 32.4) and genera (132.8 ± 28.2) were obtained from the Diet3 group, in relation to other diets. The rarefaction plot indicated that each sample was sequenced at enough depth and up to saturation to capture most of the bacterial diversity (Fig. 4A). All samples also had high good’s coverage index, ranging from 0.998 to 0.999, implying satisfactory bacterial coverage. Diets 2 and 3 had the highest (P < 0.05) alpha diversity indices in term of observed OTUs and Shannon (Fig. 4B), indicating that this group had a different spectrum of bacterial diversity when compared to other diets. Beta ordination showed distinct clustering of bacterial OTUs and PERMANOVA R-value of 0.46 and P-value of 0.032 revealed that feeding had significant impacts on gut microbial diversity (Fig. 4C).

### Microbial communities

Relative abundance at the phyla level showed that Proteobacteria was most dominant in Diet1 and Diet4 while *Firmicutes* richness was observed for Diet2 and Diet3 (Fig. 4D). At the genus level, Diet3 found to influence the colonization of more bacteria than other diets and therefore, a diverse range of genera was identified, including *Ruminococcus* (58%), *Psychrobacter* (12%), *Lachnospira* (11%), *Clostridium* sensu stricto (8%) and *Ruminoclostridium* (8%). Diet1, Diet2, and Diet4 were dominated by *Vibrio* (71%), *Staphylococcus* (44%), and *Rubritelea* (57%), respectively (Fig. 4E). Differential abundance with LDA 2.0 and above and at 0.05 level of significance identified *Ruminococcus* and *Lactobacillus* as significantly abundant genera in Diet3, and *Vibrio, Staphylococcus*, and *Rubritelea* in Diet1, Diet2 and Diet4 groups, respectively (Table 8).

**Table 8:** Distinguishing bacterial genera in different diets after feeding trial.

Genus	Diet1 (Control)	Diet2 (80PBM_10TH+10HI_)	Diet3 (85PBM_5TH+10 HI_)	Diet4 (90PBM_5TH+ 5HI_)	P value
*Vibrio*	43.08 ± 3.09	1.62 ± 0.63	1.02 ± 0.68	5.44 ± 2.2	< 0.001
*Ruminococcus*	1.89 ± 0.92	0.68 ± 0.61	16.52 ± 4.22	0.81 ± 0.07	< 0.001
*Staphylococcus*	2.34 ± 0.38	48.86 ± 9.98	2.33 ± 0.67	2.58 ± 1.78	< 0.001
*Rubritalea*	10.05 ± 1.61	11.55 ± 4.18	3.68 ± 1.08	47.02 ± 13.01	< 0.01

Each value displayed under the columns diet represents mean relative abundance with standard deviation.

### Bacterial challenge

**Figure 5:**
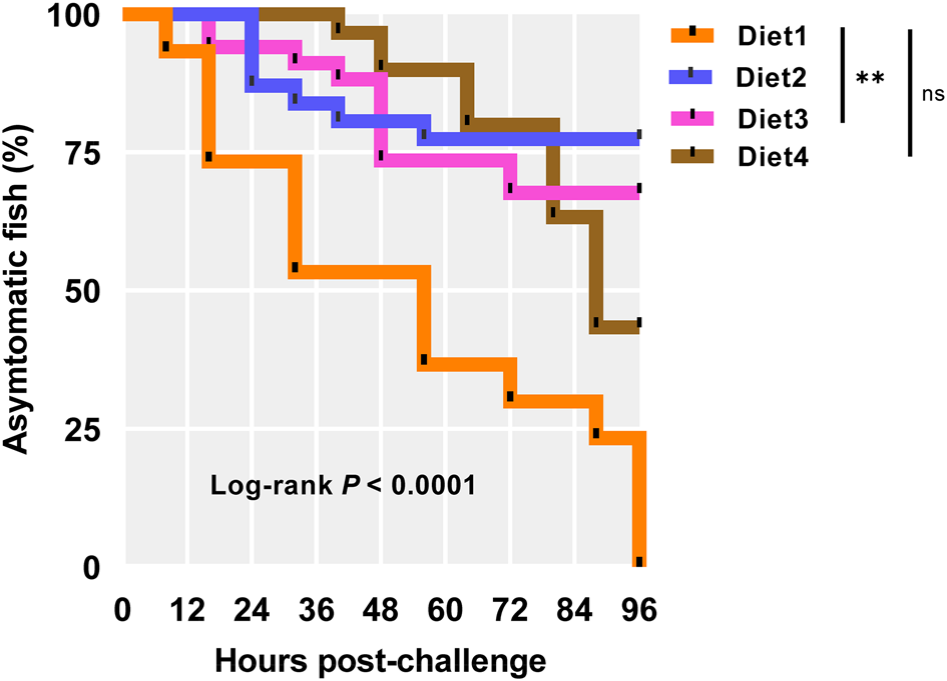
Variation in the infection rate in fish fed different levels of PBM concurrently supplemented with TH and HI larvae after 96 h post-challenge by injection with *V. harveyi*. Infection started in Control at 8 h post challenge (hpc) and while infection started at 24 hpc in 80PBM_10TH+10HI_, 16 hpc in 85PBM_5TH+10HI_ and 40 hpc in 90PBM_5TH+5HI_, respectively. Asterisks demonstrated significant variation between Control- vs 80PBM_10TH+10HI_, and 85PBM_5TH+10H_—fed fish at *P* < 0.01 (Kaplan Meyer survival method, followed by Log-rank test).

Infection rate in fish fed 80PBM and 85PBM supplemented with TH and HI larvae meal was significantly lower than the control (Fig. 5). Meanwhile, infection rate in fish fed TH and HI larvae meal supplemented 90PBM showed no significant variation than control.

## Discussion

The ability of FPH supplementation to enhance growth performance has been reported in many species^23,76–78^, however, the concurrent supplementation of TH and HI larvae meal to non-FM based in the diet of barramundi has been reported for the first time in the present study. The total substitution of FM with PBM concomitantly supplemented with TH and HI larvae improved the growth and condition factor in barramundi. Our earlier^20^ and other study by Siddik, et al.^21^ evaluating the total FM substitution with PBM resulted in poor growth performance in barramundi . Also, many other studies reported negative outcomes in the growth of a number of fish when dietary inclusion of PBM exceeded > 50%^15–17^. The ameliorative effects on growth and condition factor in the present study could be explained by the presence of greater amounts of low molecular weight peptides in TH and bioactive peptides in HI larvae. The molecular weight of peptides in TH in our earlier study showed that more than 90% of the peptides were less than 10 kda^25^ which have been reported as biologically active peptides acting as growth promoters^79–82^. The hydrolysis process has been reported to elevate chemical and functional properties of feed as well as produce free amino acids, di- and try-peptides which reach the intestine faster than intact proteins and are easily absorbed by enterocytes^76,83^. Also, inclusion of insect meals at lower levels has been recommended by many researchers to promote the growth and health. For instance, Chaklader, et al.^13^ reported a significantly higher growth performance in barramundi fed a 10% full-fat HI larvae supplemented PBM diet. Caimi, et al.^40^ recommended to include 18.5% HI larvae meal in the diet of Siberian sturgeon, *Acipenser baerii* without affecting antioxidant response, liver and gut health.

The taste and appearance of cooked flesh for the consumer is influenced by the lipid composition in the edible part of fish fillets^84,85^. The higher lipid levels in PBM fed fish in the current study was similar to the reported lipid content in rainbow trout^86^ and tench^15^, however, contradict the results of^24^ who found lower levels of lipid in barramundi fed PBM based diets supplemented with TH. The higher fillet lipid content in this study could be due to higher levels of lipid content in the added full-fat HI larvae meal. The unchanged moisture, protein and ash content is similar to previous studies examining barramundi fed PBM based diets^21,24^.

In this study, total SFA content increased in fish fed all inclusion levels of PBM concurrently supplemented with TH and HI larvae due to a rise in lauric acid (C12:0), myristic acid (C14:0), palmitic acid (C16:0), and stearic acid (C18:0), and confirming that fatty acid composition of fish is generally a reflection of the fatty acids composition in the diets^87^. HI larvae meal contain higher levels of lauric acid, causing an increase in lauric acid content in rainbow trout, *Oncorhynchus mykiss* Walbaum^87^ and juvenile jian carp, *Cyprinus carpio* var. Jian^88^ when fed HI larvae based-diets. Also, a rise in palmitic acid and stearic acid levels led to an elevated total SFA in the muscle of barramundi fed PBM based diets supplemented with various FPH^25^. However, lauric acid, palmitic acid, and stearic acid were relatively low in the muscle of fish fed TH and HI supplemented PBM than the dietary concentrations, perhaps indicating the utilization of these SFA for energy production. Similar to total SFA content, total MUFA content improved in the muscle of fish fed PBM diets, supplemented with TH and HI, similar to the muscle fatty acids of barramundi fed FPH supplemented PBM based diets^25^. This increase is mainly due to the higher proportion of MUFA content in the PBM based diets. Meanwhile, concurrent TH and HI supplemented PBM based-diets contained higher PUFAs content than the control, causing a corresponding augmentation in total PUFA content in the muscle of fish. However, feeding barramundi with exclusive levels of PBM reduced the total PUFA particularly n-3 PUFA in the muscle of barramundi^21^. The amelioration of PUFA content in the present study could be due to the supplementation of TH and HI as they contain a higher amount of PUFA than PBM. Similarly, the modulatory effect of FPH on lipid accumulation, lipid metabolism, and fatty acid composition has been reported in barramundi^25^, turbot, *Scophthalmus maximus*^89^ and some other animals (mice/rats)^90,91^. The FA results suggest that total replacement of FM with PBM supplemented with TH and HI improved the fillet FA composition, manifested by an increased level of MUFA and PUFA content. Such changes suggest that barramundi produced from feeds examined in this study would be beneficial to human health since MUFA and PUFA content are highly associated with reducing the risk of cardiovascular and neurological disease^92^.

Besides the fatty acid profile, post-harvest fillet characteristics such as texture and colour are affected by dietary modification which are subsequently reflected in consumer acceptability and thus market demand. However, previous no information was available on the effects of total substitution of FM with PBM on the post-harvest quality of barramundi fillets. High inclusion of meat meal (40% or more) at the expense of FM did not adversely affect the organoleptic quality of 66 days post-feeding pond-reared barramundi muscle^59^ which was similar to the current study. Whereas, 100% substitution of FM with PBM negatively influenced the sensory characteristics of female tenches, *Tinca tinca*^60^.

Histological damages of immune-sensitive organs including liver, kidney, and spleen could be easily assessed when host species are fed non-FM diets. For example, total substitution of FM with PBM resulted in lipid droplets and multifocal necrosis in the liver of barramundi^13,20^. Similarly, higher replacement of FM with PBM (50–70%) and animal protein blend containing PBM, shrimp meal and spray-dried blood meal (20–80%) induced hepatocytes steatosis in juvenile hybrid grouper, *Epinephelus fuscoguttatus* ♀ × *Epinephelus lanceolatus* ♂^8,93^. However, supplementation of TH and HI larvae meal appear to prevent such organ damage in the current study. FPH supplementation ameliorates the alternative protein quality and reduces the lipid accumulation coupled with increasing lipid metabolism in fish^89^ and other animals. Also, the presence of chitin and its derivatives in HI larvae have been reported to boost the hydrolysis of lipoproteins and triglycerides, coupled with reducing the synthesis of fatty acids in the liver of fish and other animals ^88,94,95^. Besides chitin, lauric acid (C12:0), one of the main fatty acids in HI larvae could also prevent liver damage induced by PBM in the current study since animals can quickly oxidize lauric acid after consumption rather than being stored in liver^96^. A similar effect was observed by Kumar, et al.^97^ who reported biliary duct hyperplasia in the liver of rainbow trout fed HI larvae oil, indicating more release of bile to facilitate lipid digestion.

Evaluating intestinal morphological structure is also important to understand the potential effects of alternative protein on any fish health, as such evaluation is reported to be highly correlated with nutrient assimilation and immunological function^98^. An improvement in microvilli density intestine indicates elevated enterocyte absorptive surface area^99^ and neutral mucins number in the intestine have been linked to the protection of the intestinal epithelium by lubricating, trapping and eradicating opportunistic pathogens^100,101^, prevent proteolytic damages and promoting enzymatic digestion and functionality of the gastric glands^102–104^. In this study, barramundi fed different levels of PBM with TH and HI larvae meal demonstrated a higher microvilli density and higher levels of neutral mucins than the Control, indicating more uptake and transportation of amino acids and free fatty acids^105^. Such effects might modulate the growth performance and disease resistance via improving the serum immune response. This could be due to the concurrent effect of TH and HI to confer a relatively higher benefit than when TH was used alone with 75 and 90% PBM in barramundi^24^. Similarly, HI larvae meal supplementation alone with 90% PBM did not influence villus height and neutral mucins in barramundi^13^. Regardless of TH supplementation, antimicrobial peptides, chitin, and lauric acid reported in HI larvae might have played a further key role in improving intestinal health since a number of recent studies have reported probiotic effects of HI larvae meal in rainbow trout^106–108^.

Goblet cells synthesize biologically active substances and other numerous defensive molecules, generally known as mucus, which have been reported to exert an important role in both the innate and acquired immune systems in fish. Goblet cells enumeration in the skin revealed by AB-PAS showed that total substitution of FM with PBM supplemented with TH and HI larvae did not affect the number of goblet cells. However, supplementation of various hydrolysates with PBM improved the number of goblet cells in terms of acidic mucins in barramundi^25^.

Valuable information on internal organs, nutritional status, and metabolic state can be achieved through the investigation via a panel of serum biochemical assays. Dietary incorporation of exclusive levels of alternative animal protein, in particular PBM, has been reported to have negative impacts on the activity of fish liver enzymes such AST, ALT and GLDH^8,13^, which leak into the blood at abnormal levels when there is liver cell damage. In the present study, such a negative impact was not observed in the measured activity of ALT, GGT, and GLDH, indicating that supplementation of TH and HI larvae meal could prevent liver damage caused by higher inclusion levels of PBM. Total bilirubin, which at high levels can be associated with kidney damage, was significantly lower in fish fed TH and HI larvae supplemented PBM diets, suggesting no adverse effects of total substitution of FM with PBM on kidney function. Similar effects were observed in barramundi fed a TH supplemented PBM diet^25^. Serum TP is usually more stable in fish in well-nourished conditions^109^ and elevation in levels is indicative of stronger innate immunity in fish^110^. A significant increase in the TP levels in fish fed concomitant TH and HI larvae supplemented PBM diets indicates an improvement in the immune system. However, TH and HI larvae meal supplementation separately with PBM did not influence the serum TP in barramundi^25^. Other unchanged biochemical assays including urea, creatinine, and cholesterol, were within the reported healthy ranges for barramundi^25^.

It is well established that FPH supplementation has been reported to improve diet quality^24,111^, coupled with immunostimulating properties due to the presence of biologically active di- and tripeptides and other oligopeptides^112,113^. Separate supplementation of FPH or insect larvae with PBM improved the immune response such as lysozyme and bactericidal activity in barramundi^13,24^ but combined supplemental effects on barramundi immunity is reported here for the first time. In the present study, different levels of PBM supplemented with TH and HI larvae modulated the lysozyme and bactericidal activity of barramundi. Similarly, supplementation of FPH and crustacean hydrolysate have been reported to improve the nutritional quality of alternative protein source including PBM and plant protein with concomitant modulatory effects on immune responses such as lysozyme and complement activity in barramundi^24^ and European sea bass, *Dicentrarchus labrax*^114^. The modulatory effects of TH supplemented PBM diets could be due to a higher proportion of low molecular weight peptides in TH as earlier studies confirmed immune-stimulating properties of medium size-bioactive peptides (3000 Da > Mw > 500 Da)^115,116^. Besides the beneficial effects of TH, HI larvae supplementation could further boost the immunity of barramundi since earlier reports showed that HI larvae contain antibacterial peptides and some polysaccharides such as silkose or dipterose which, along with chitin, play an important role in modulating the immune response. Elevated immune response was reported in gilthead seabream, *Sparus aurata* L.^117^ and common carp, *Cyprinus carpio*^118^ when fed low levels of crustacean derived chitin which was not analysed in HI larvae meal in the present study. Similar with the present findings, an upsurge in lysozyme and bactericidal activity was reported in barramundi fed 10% HI larvae with a PBM based diet^13^ and also enhanced lysozyme, complement 3 and complement 3 was found in black carp, *Mylopharyngodon piceus* fed 25 g kg^−1^ maggot meal in a FM-based diet^119^.

Reactive oxygen species (ROS) including superoxide anion (O^2−^), hydrogen peroxide (H_2_O_2_) and hydroxyl radical (OH) are essential by-products of phagocytic processes, causing impairment of DNA and injury to lipids, proteins and nucleic acids membranes, or even death if there is no equilibrium between ROS production and antioxidant defence mechanisms. Like other terrestrial animals, fish have a similar manner of enzymatic and non-enzymatic antioxidant defence mechanisms to protect themselves from oxidative damage^120^. Radical scavenging enzymes particularly CAT protect cells from oxidative damage by converting hydrogen peroxide into water^121^. In the present study, CAT activity both in serum and the liver was not affected by PBM inclusion supplemented with TH and HI larvae, suggesting that total replacement of FM with PBM supplemented with TH and HI larvae did not cause potential damage in the liver of barramundi. The present results agree with the findings of Siddik, et al.^22^ who reported no alteration of CAT activity in the serum of barramundi when fed TH supplemented PBM diets. However, feeding animal protein blends including PBM, shrimp meal, and spray-dried blood meal impaired the liver health of hybrid grouper, *Epinephelus fuscoguttatus*♀ × *Epinephelus lanceolatus*♂^8^. No impairment in the liver of barramundi fed PBM based diets reported here can be explained by the supplementation of TH and HI larvae since many studies have been reported the presence of antioxidative capacity of FPH and insect larvae.

Due to the advent of NGS technologies, knowledge of intestinal microbial ecosystem alteration in response to dietary modification has improved. High bacterial diversity in the intestine of fish fed 80PBM_10TH+10HI_ and 85PBM_5TH+10HI_ than the control may indicate a healthier gut since several studies have reported a number of beneficial effects of the rich bacterial community such as out-competition pathogens for nutrients and colonization and consequently resisting pathogen invasion and intestinal infection^108,122,123^. The possible reason for the highest bacterial richness may be the supplementation of HI larvae rich in chitin and lauric acid content which are similar to the bacterial diversity of rainbow trout, *Oncorhynchus mykiss*^106–108^, and other animals such as laying hens^124^ fed HI larvae meal-based diets. The unchanged bacterial diversity in 90PBM_5TH+5HI_ with respect to Control could be due to the highest replacement of FM protein with PBM. The same effect is supported by an earlier study in our laboratory with no changes in intestinal bacteria diversity of barramundi fed 90PBM supplemented with FPH^22^. In agreement with the earlier studies on barramundi, metagenomics results revealed that *Proteobacteria*, *Firmicutes,* and *Bacteroidetes* were the dominant phyla in all groups^22,125,126^. Indeed these phyla constitute the “core gut microbiota” constituting up to 90% in the intestine of different marine water fish species^127–129^. There is little information concerning the effects of dietary replacement of FM with alternative protein sources on the intestinal gut microbiota of barramundi. However, the dietary substitution of FM with a mix of terrestrial animal and plant proteins did not influence the intestinal microbiota of barramundi^126^ whereas a recent study^22^ found a modulatory effect of fermented PBM along with supplementation of TH on the intestinal microbial community. In this study, *Proteobacteria* abundance was improved in the intestine of fish fed Control and 90PBM_5TH+5HI_ whilst *Firmicutes* were enriched in 80PBM_10TH+10HI_ and 85PBM_5TH+10HI_ fed groups. At the genus level, *Ruminococcus* and *Lactobacillus,* under *Firmicutes* phylum, were significantly more abundant in fish fed 85PBM_5TH+10HI_. The *Ruminococcus* genus is highly associated with the degradation of indigestible carbohydrate ingredients particularly resistant starch and dietary fibres, thereby contributing to a more efficient energy utilization of feed and this genus also plays an important role in the fermentation of dietary fibre, the subsequent butyrate end product is reported to influence the intestinal health of the host^130–133^. Also the genera of lactic acid bacteria (LAB), in particular *Lactobacillus*, are beneficial microorganisms commonly used as probiotics for fish and other vertebrates^134^ due to an ability to create biofilms by producing bacterial compounds (lactic acid hydrogen peroxide, and bacteri-ocins or biosurfactants) that can prevent the adherence of pathogens to the intestinal surface^135^. Similar to the present study, dietary inclusion of partially defatted HI larvae was reported to enrich the LAB in the intestine of rainbow trout, *Oncorhynchus mykiss*^106,107^. The presence of chitin and MCFA, particularly lauric acid, in HI larvae meal were reported to have a modulatory effect on LAB, as reported by the same authors. In addition, TH might have influenced the LAB abundance since FPH is a good source of nitrogen serving as a good medium for bacterial growth^76^. However, the lower abundance of *Vibrio* in test diets in the present study could be due to the inclusion of HI larvae irrespective of TH supplementation. Similarly, the abundance of *Vibrio* decreased in the intestine of zebrafish when fed various levels of HI larvae meal^136^. The presence of chitin, lauric acid, and recently extracted novel antimicrobial peptides of HI larvae meal are active against Gram-negative and Gram-positive bacteria^13,44,106^ which could have reasonably negatively influenced the abundance of *Vibrio* in the current study. The *Staphylococcus*, and *Rubritelea* were abundant in control, 80PBM_10TH+10HI_ and 90PBM_5TH+5HI_ groups. The presence of *Staphylococcus* is common in the gastrointestinal tract of fish, have been documented in several studies over the last decade^137,138^.

Supplementation of 3% anchovy and giant squid hydrolysates in the diet of European seabass, *Dicentrarchus labrax* showed bactericidal and bacteriostatic activities against a number of fish pathogenic bacteria including *V. parahaemolyticus*, *V. harveyi* and *P. damselae* subsp. *piscicida*, and *V. anguillarum*^139^. A number of previous studies supplementing FPH have also proven the modulation of disease resistance against pathogenic bacteria in barramundi^23,24^, red sea bream, *Pagrus major*^80,82,140^ and European sea bass, *Dicentrarchus labrax*^141^. In the present study, the improved infection rate against *V. harveyi* in the test diets could be due to the presence of bioactive peptides in FPH. Also improved infection rate may be due to the presence of low molecular weight peptides (< 6500 Da) described in our earlier study in TH^25^ with possible antibacterial properties^142^, as supported by the improved lysozyme and bactericidal activity of barramundi fed TH supplemented PBM. Some peptides, in particular lactoferrin-derived peptides, have the ability to damage the bacterial membrane of different species and strains of *Vibrio*^143^, and this remains to be further studied. Besides the role of TH, the presence of antibacterial peptides in HI larvae meal has been demonstrated to have an inhibitory response against Gram-positive bacteria, Gram-negative bacteria and fungus^44^ and this may have also influenced the infection rate. In our earlier study, supplementation of 10% HI larvae meal with 45% PBM modulated the disease resistance in response to a two-week challenge with *V. harveyi*^13^. However, the specific role of antibacterial peptides in TH and HI larvae in boosting the immune response of fish need to be further explored.

In summary, concurrent supplementation of 10 and/or 5% TH and HI larvae can facilitate the total replacement of FM with PBM in barramundi, including an improved growth performance and biometry indices, and improved microvilli density and neutral mucin levels in intestine. Fatty acid profiles such as MUFA and PUFA improved in TH and HI supplemented PBM fed barramundi with no significant effects on all tested post-harvest fillet quality characteristics. No histopathological changes in liver, kidney and spleen further proved the ability of TH and HI supplementation to hamper the negative effects previously reported to be caused by dietary animal protein ingredients. Whilst serum biochemical assays, particularly total bilirubin and total protein, improved in TH and HI larvae supplemented PBM groups, antioxidant activities in the serum and liver were unaffected. Concurrent supplementation of TH and HI larvae demonstrated bioactivity via modulating lysozyme and bactericidal activity which may elucidate higher survival rates in response to *V. harveyi* infection. Also, bacterial diversity on 80PBM_10TH+10HI_ and 85PBM_5TH+10HI_ increased but the presence of *Vibrio* decreased in barramundi fed PBM based diets concurrently supplemented with TH and/or HI larvae meal.

## Data Availability

The amplicon sequence data in fastq format has been deposited to National Centre for Biotechnology Information (NCBI) under the BioProject accession number PRJNA660066.
